# Hereditary Optic Neuropathy Associated with a Novel MT-RNR1 Mutation: A Case Report

**DOI:** 10.1159/cop/acaag016

**Published:** 2026-09-08

**Authors:** Asma Bilgasem, Haoran Charles Li, Jonathan A. Micieli

**Affiliations:** a Temerty Faculty of Medicine, University of Toronto, Toronto, ON, Canada; b Faculty of Medicine, University of British Columbia, Vancouver, BC, Canada; c Kensington Vision and Research Centre, Toronto, ON, Canada; d Department of Ophthalmology and Vision Sciences, University of Toronto, Toronto, ON, Canada; e Division of Neurology, Department of Medicine, University of Toronto, Toronto, ON, Canada

**Keywords:** Hereditary optic neuropathy, MT-RNR1, m960delC, Case report

## Abstract

**Introduction:**

Hereditary optic neuropathies are mitochondrial disorders characterized by retinal ganglion cell degeneration and optic nerve dysfunction. Variants in MT-RNR1, a gene classically associated with aminoglycoside-induced and nonsyndromic sensorineural hearing loss, are increasingly recognized in hereditary optic neuropathy. We report the first case of hereditary optic neuropathy associated with the novel MT-RNR1 m.960delC variant, expanding the known genetic spectrum of this emerging mitochondrial optic neuropathy phenotype.

**Case Presentation:**

A 61-year-old woman presented with bilateral optic neuropathy characterized by optic disc pallor out of proportion to cupping, diffuse retinal nerve fiber layer and ganglion cell layer thinning, and bilateral visual field abnormalities. Best-corrected visual acuity was 20/30 in the right eye and 20/20 in the left eye, with intraocular pressures within the normal range. Gonioscopy demonstrated open angles bilaterally. Visual field testing demonstrated nonglaucomatous defects, and laboratory investigations were unremarkable. MRI of the orbits and brain with gadolinium was normal. Mitochondrial genetic testing identified the novel MT-RNR1 m.960delC variant at 87.2% heteroplasmy. The overall phenotype was consistent with hereditary optic neuropathy associated with mitochondrial dysfunction.

**Conclusion:**

This case expands the spectrum of MT-RNR1-associated hereditary optic neuropathy through identification of the novel m.960delC variant. The proximity of m.960delC to the previously reported m.956delC variant raises the possibility of a functionally important region within MT-RNR1. These findings further support MT-RNR1 as an emerging candidate gene in hereditary optic neuropathy, with important implications for genetic testing and disease recognition.

## Introduction

Hereditary optic neuropathies are mitochondrial disorders characterized by degeneration of retinal ganglion cells and progressive bilateral vision loss. While the majority of cases are associated with OPA1-related dominant optic atrophy or the three primary mitochondrial DNA mutations causing Leber hereditary optic neuropathy (LHON), the genetic spectrum of hereditary optic neuropathy continues to expand with the identification of additional mitochondrial and nuclear variants associated with optic nerve degeneration [1–3].

Variants in MT-RNR1 are classically associated with aminoglycoside-induced and nonsyndromic sensorineural hearing loss, particularly the m.1555A>G mutation [4]. However, its role in optic neuropathy is only beginning to be recognized. Secondary MT-RNR1 variants have been identified in LHON and other mitochondrial disorders [5–7], and novel MT-RNR1 variants were recently reported in 2 patients with hereditary optic neuropathy [8]. These observations suggest that MT-RNR1-associated optic neuropathy is a rare but emerging clinical entity.

Given the rarity of MT-RNR1-associated optic neuropathy, the continued identification of novel mitochondrial variants, and the potential for atypical clinical presentations, reporting well-characterized cases remains important to refine genotype-phenotype associations and improve disease recognition. Here, we describe a patient with hereditary optic neuropathy associated with the novel MT-RNR1 m.960delC variant, which to our knowledge has not previously been reported in association with optic nerve disease. The authors have completed the CARE Checklist for this case report, which is attached as online supplementary material (for all online suppl. material, see https://doi.org/10.1159/cop/acaag016).

## Case Presentation

A 61-year-old woman was referred in August 2022 for evaluation of advanced glaucoma in both eyes (OU). The patient reported no subjective visual complaints. Her medical history was significant for a 2-year history of type 2 diabetes mellitus and ovarian cancer. She was not taking any medications at presentation and had no known family history of glaucoma or vision loss.

At initial evaluation, best-corrected visual acuity was 20/30 in the right eye (OD) and 20/20 in the left eye (OS), with intraocular pressures of 11 mm Hg OU. She had previously been started on topical glaucoma therapy (travoprost/timolol once nightly OU) for presumed advanced normal-tension glaucoma. However, given the subsequent concern for a nonglaucomatous hereditary optic neuropathy and consistently normal intraocular pressures without evidence of progression, topical therapy was later discontinued.

Over longitudinal follow-up from August 2022 through April 2026, visual acuity and visual fields remained stable, and intraocular pressures consistently remained within the normal range. Notably, despite the apparent severity of structural findings at presentation, there was no clear evidence of progression.

On examination, she demonstrated enlarged cup-to-disc ratios bilaterally with optic disc pallor. She had no prior history of optic nerve abnormalities or visual dysfunction. Optical coherence tomography (OCT) obtained on March 1, 2024 (Fig. 1a), showed diffuse and symmetric thinning of the retinal nerve fiber layer (RNFL), with average thickness of approximately 50 µm OU. Humphrey visual field testing (24-2) demonstrated scattered, nonspecific depression on pattern deviation analysis without an arcuate defect, nasal step, paracentral scotoma, or other characteristic glaucomatous pattern (Fig. 1b). Gonioscopy demonstrated open angles bilaterally.

**Fig. 1.**
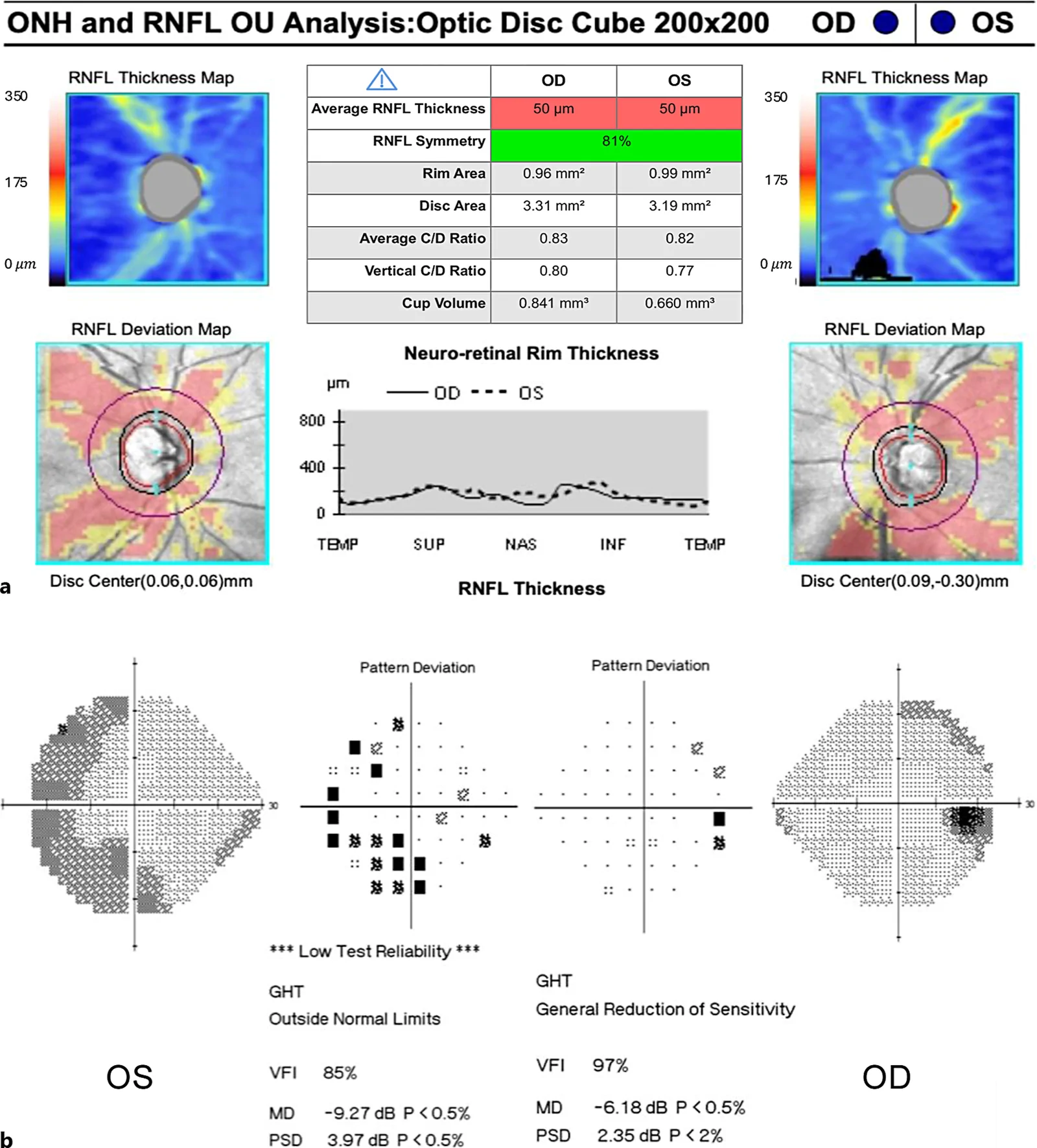
OCT and Humphrey visual field testing at initial evaluation. **a** Peripapillary RNFL thickness maps demonstrating diffuse bilateral thinning with reduced average RNFL thickness. **b** Humphrey visual field (24-2) demonstrating scattered, nonspecific depression on pattern deviation analysis without an arcuate defect, nasal step, or other characteristic glaucomatous pattern. OCT, optical coherence tomography; RNFL, retinal nerve fiber layer.

The combination of optic disc pallor out of proportion to cupping, diffuse structural loss, and nonglaucomatous visual field abnormalities raised concern for a nonglaucomatous optic neuropathy. The optic nerve appearance was not consistent with congenital hypoplasia and the patient confirmed her optic nerve was reportedly normal in appearance in her early teenage years. Laboratory studies, including complete blood count, vitamin B12, and folate levels, were within normal limits. MRI of the orbits and brain with gadolinium was normal. Mitochondrial genetic testing subsequently identified the MT-RNR1 m.960delC variant at 87.2% heteroplasmy.

At most recent follow-up in April 2026, visual acuity remained stable, and intraocular pressures were 11–12 mm Hg without treatment. Repeat OCT demonstrated persistent diffuse thinning of the RNFL and ganglion cell layer (GCL), with average RNFL thickness of 50 µm OU and GCL thickness of 54 µm OU, without evidence of progression. Visual field testing also remained stable over longitudinal follow-up without evidence of progressive change (Fig. 2a–c). The overall clinical phenotype, including bilateral optic disc pallor, diffuse symmetric RNFL and GCL thinning, bilateral visual field abnormalities, and identification of the novel MT-RNR1 m.960delC variant, was consistent with hereditary optic neuropathy associated with mitochondrial dysfunction.

**Fig. 2.**
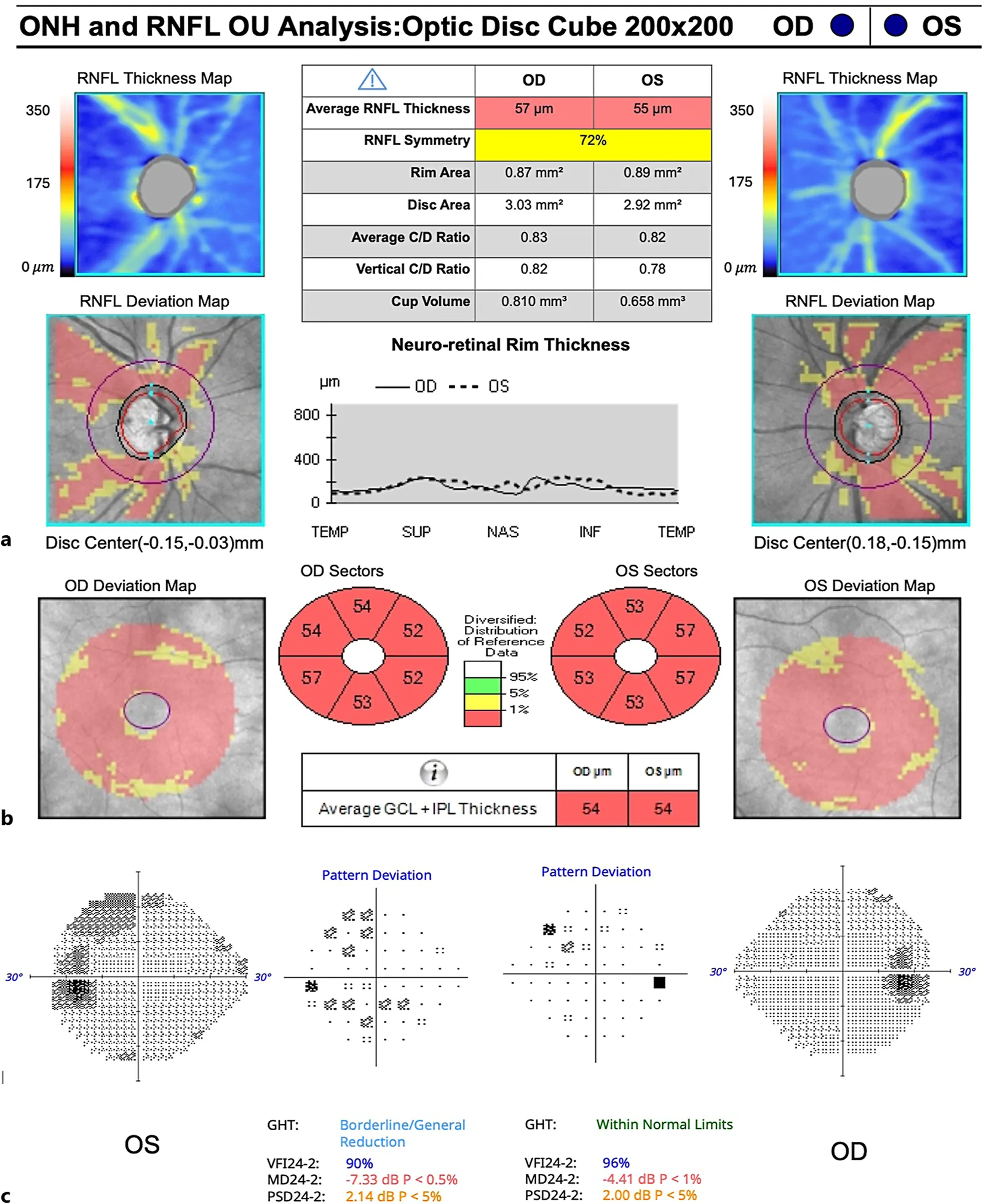
OCT and Humphrey visual field testing at follow-up. **a** Peripapillary RNFL thickness maps demonstrating persistent diffuse bilateral thinning with reduced average RNFL thickness. **b** Macular GCL analysis demonstrating symmetric global thinning bilaterally. **c** Humphrey visual field (24-2) demonstrating stable, nonspecific depression on pattern deviation analysis without a characteristic glaucomatous pattern. OCT, optical coherence tomography; RNFL, retinal nerve fiber layer; GCL, ganglion cell layer.

## Discussion

We report a case of hereditary optic neuropathy associated with a novel MT-RNR1 variant, m.960delC, identified at 87.2% heteroplasmy. To our knowledge, this variant has not previously been reported in association with optic nerve disease. While the clinical phenotype overlaps with recently described MT-RNR1-associated optic neuropathy cases [8], the primary novelty of this report is the identification of a previously undescribed MT-RNR1 deletion variant associated with hereditary optic neuropathy.

The MT-RNR1 gene encodes the mitochondrial 12S ribosomal RNA, an essential structural component of the small mitochondrial ribosomal subunit involved in mitochondrial protein translation and oxidative phosphorylation. Retinal ganglion cells are highly dependent on mitochondrial energy production, making them particularly vulnerable to mitochondrial dysfunction. Impaired mitochondrial translation and oxidative phosphorylation may result in reduced ATP production, increased oxidative stress, and retinal ganglion cell degeneration – mechanisms central to hereditary optic neuropathies [1, 9, 10].

Although hereditary optic neuropathies are classically associated with OPA1-related dominant optic atrophy and the three primary LHON mitochondrial DNA mutations, additional nontraditional mitochondrial variants continue to expand the genetic spectrum of disease [1]. MT-RNR1 variants are best known for their association with aminoglycoside-induced and nonsyndromic sensorineural hearing loss, particularly the m.1555A>G variant, which has been shown to alter mitochondrial ribosomal RNA structure and increase susceptibility to aminoglycoside toxicity [4, 11]. Prior studies have also identified secondary MT-RNR1 variants in LHON families and have associated MT-RNR1 variation with broader neurologic phenotypes including epilepsy and intellectual disability, supporting the biologic importance of this gene in mitochondrial disease [5–7].

Recently, novel MT-RNR1 variants were reported in 2 patients with hereditary optic neuropathy characterized by bilateral temporal optic disc pallor, RNFL thinning, and ganglion cell complex loss [8]. Both patients demonstrated relatively preserved visual acuity despite structural evidence of optic nerve injury and had a mild, slowly progressive clinical course compared with classic LHON. Similar to these previously reported cases, our patient demonstrated diffuse ganglion cell loss with preserved central visual acuity despite clear structural abnormalities on OCT, supporting MT-RNR1 as a candidate gene in hereditary optic neuropathy.

In our patient, the m.960delC variant was identified following exclusion of nutritional, inflammatory, demyelinating, compressive, retinal, and toxic causes of optic neuropathy, including a normal MRI of the orbits and brain with gadolinium. Particularly noteworthy is its proximity to the previously reported m.956delC variant, which has been identified in mitochondrial disease cohorts and evaluated in structural modeling studies of mitochondrial ribosomal RNA [5]. Both variants represent single-nucleotide deletions occurring within a four-nucleotide span of MT-RNR1. The occurrence of two independent deletion variants in such close proximity raises the possibility that this region represents a structurally or functionally important domain. Although causality cannot be inferred from isolated observations, this regional clustering may represent an emerging genotype-phenotype signal warranting particular attention in future studies.

As MT-RNR1 encodes a ribosomal RNA rather than a protein-coding gene, there are no established in silico prediction tools capable of reliably assessing pathogenicity of these variants [12]. Definitive classification of m.960delC therefore remains challenging and will require identification of additional affected individuals and further characterization of variants in this region.

While heteroplasmy thresholds have been established for some mitochondrial disorders, the relationship between heteroplasmy burden and phenotypic expression in MT-RNR1-associated optic neuropathy remains poorly understood. The high heteroplasmy level of 87.2% is nonetheless supportive of a pathogenic role when considered alongside the compatible clinical phenotype, exclusion of alternative etiologies, and emerging evidence linking MT-RNR1 variation to hereditary optic neuropathy [5–8].

In conclusion, this case expands the spectrum of MT-RNR1-associated hereditary optic neuropathy and further supports MT-RNR1 as a candidate gene in mitochondrial optic nerve disease. The novel m.960delC variant has not previously been reported in association with optic nerve disease, and its proximity to m.956delC raises the possibility of a functionally important region within MT-RNR1. The CARE checklist has been completed by the authors for this case report, attached as online supplementary material.

## Statement of Ethics

This retrospective review of patient data did not require ethical approval in accordance with local/national guidelines. All patients provided written informed consent for the publication of this case report and accompanying images.

## Conflict of Interest Statement

The authors declare no conflicts of interest.

## Funding Sources

This study was not supported by any sponsor or funder.

## Author Contributions

Asma Bilgasem: writing – original draft, methodology, validation, investigation, preparation, and visualization. Haoran Charles Li: validation, reviewing, and editing. Jonathan A. Micieli: supervision, conceptualization, validation, reviewing, and editing.

## Data Availability

The data supporting this study’s findings are not publicly available due to privacy and security reasons but can be obtained from the corresponding author upon reasonable request.
